# Empowerment of parents of infants with congenital heart disease after rapid genome sequencing: April 11, 2025, revised June 20, 2025

**DOI:** 10.1007/s12687-025-00813-3

**Published:** 2025-07-14

**Authors:** Rita M. Cheney, Gabrielle C. Geddes, Sara M. Fitzgerald-Butt

**Affiliations:** Department of Medical and Molecular Genetics, Indiana University School of Medicine, Indianapolis, IN USA

## Abstract

**Background:**

Despite rapid genome sequencing (rGS) being utilized as a first-tier genetic test for infants with congenital heart disease (CHD), little is known about its impact on parental empowerment.

**Methods:**

To address this gap, parents of infants with CHD (≤ 1 year old at the time of inpatient rGS) were asked to participate in an online survey, which measured empowerment using an adapted version of the Genomic Empowerment Scale (GEmS). The scale consists of four subscales that measure emotional management, meaning making, seeking information and support, and implications and planning surrounding a child’s diagnosis. Subscale scores were standardized for comparison and coded as above (+) or below (-) the mean. Based on the standardized score pattern (+/-) in each subscale, an empowerment profile was assigned to each participant. Empowerment profiles were analyzed for trends based on CHD type (left ventricular outflow tract obstruction (LVOTO) vs. non-LVOTO), genetic test result type, and number of genetics visits.

**Results:**

The most common empowerment profile was the ‘Engaged but Worried Planner’ (15/37 = 41%). This empowerment profile was more common in parents of infants with non-LVOTO CHD (73.3%) than those with LVOTO CHD (26.7%). Conversely, there was little difference in empowerment profile type between rGS result type. Parents whose child had ≤ 3 genetics visits displayed the ‘Engaged but Worried’ profile most often, whereas those with ≥ 4 visits had more even distributions between profiles.

**Conclusion:**

Understanding empowerment profiles in this population may help guide practitioners to empower parent decision-making, emotional management, and planning for the future of their child.

**Supplementary Information:**

The online version contains supplementary material available at 10.1007/s12687-025-00813-3.

## Introduction

The growing use of rapid genome sequencing (rGS) as the first line genetic testing in pediatric and neonatal intensive care units (ICUs) (Manickam et al. 2021) has prompted the need to better understand the psychosocial status of families with critically ill children who undergo this broad testing. Infants with congenital heart disease (CHD) make up a significant portion of the pediatric critically ill population, given that CHD is prevalent in 1% of live births, or approximately 40,000 births per year (Liu et al. 2019). As the rate of rGS in the setting of CHD in pediatric and neonatal ICUs continues to rise, it becomes worthwhile to investigate how this testing impacts patients and families, especially parents.

One such method for analyzing the psychosocial experience of parents whose child has undergone rGS for CHD is through the lens of a healthcare empowerment framework (McConkie-Rosell et al. 2022). Empowerment has previously been defined as a measure of belief that patients and/or their families have a level of control and hope over complex medical situations (McAllister et al. 2008, 2011a). These studies describe four pillars of empowerment, including informed decision-making, knowledge and understanding, ability to effectively navigate health systems, and future orientation for oneself and family members (McAllister, Dunn, McAllister et al. 2008, 2011a, b). This is similar to a more recent study that applied empowerment to genomic testing, in which the authors defined empowerment as both a process and an outcome; one that involves learning new information, building skills, accessing resources, and finding support from others with similar experiences (McConkie-Rosell et al. 2019). This group created the Genomic Empowerment Scale (GEmS) (McConkie-Rosell et al. 2019), meant to measure empowerment of parents of genetically undiagnosed children by tapping into the factors of meaning-making, emotional management, seeking information, and planning (McConkie-Rosell et al. 2019). While many tools have previously been used to assess clinical genetics services (i.e., genetic counseling, genetic testing), including the Genetic Counseling Outcomes Scale, the Genomic Outcome Scale, the Genomic Knowledge Scale (Grant et al. 2019; Langer et al. 2017; McAllister, Wood, Dunn, Shiloh, & ToMcAllister et al. 2011a, b), these scales all lack assessment of at least one aspect of empowerment. Understanding empowerment allows for a more complete analysis of the spectrum of complex psychosocial experiences of parents with a child in neonatal/cardiovascular ICU settings.

Several studies have shown the involvement of genetic counselling (pre- and post-) significantly increased patient empowerment in various cohorts, as well as improved knowledge about genetics services and practical application of information (Vlaskamp et al. 2021). Additionally, use of clinical genetics services has demonstrated an impact on patient perceived control and decision-making capabilities (McAllister et al. 2008). Understanding empowerment in parents of infants with CHD is important to determine how it impacts the way in which parents take part in the medical experience of their critically ill child which may be influenced by several different medical and psychosocial variables.

Genetics providers are often involved in the medical care of infants with CHD in ICU settings as CHD has a genetic etiology which when identified in the neonatal period can impact surgical and medical management. On the other hand, the genetic etiology is often not identifiable due to our limited understanding outside of monogenic disease mechanisms (Cowan and Ware 2015). Therefore, it is important to understand the utility of rGS for patients and families beyond just the medical management changes.

Cakici et al., found that parents of infants admitted to ICUs reported positive perceived benefits and personal utility of rGS and rapid exome sequencing (rES) after return of genetic testing results, regardless of whether the results were diagnostic or not. These positive perceived outcomes were related to the process of informed consent, understanding genetic test results, and absence of decisional regret (Cakici et al. 2020), highlighting the importance of the psychosocial benefits received from rGS/rES.

At our institution, genetic services for infants with congenital heart disease generally commence while the infant is in the hospital with a physical exam and evaluation performed by a geneticist. This is then immediately or at a separate time, based on availability of parents, followed by a genetic counselor discussing the method of genetic testing, possible test result types, limitations of genetic testing, and discussion of whether the family would like to receive secondary findings. When genome sequencing results are received, they are communicated to the family in different manners depending on the type of result and whether the patient is still in the hospital ranging from in-person disclose of results to a phone call by a genetic counseling assistant. Regardless of how the disclosure is made, all patients/parents are all offered a follow-up appointment with a genetic counselor approximately two months post-discharge to review results and their implications in addition to recurrence risk and recommendations for future pregnancies and an appointment with a CV geneticist at approximately nine to twelve months of age to evaluate development and assess need for additional genetic testing and/or reanalysis.

In addition to identifying the empowerment profiles of parents of infants with CHD who have had rGS, in this study we sought to investigate whether the following clinical characteristics impacted parental empowerment profiles. As parents of infants with left ventricular outflow tract obstruction (LVOTO) CHDs (which includes hypoplastic left heart syndrome, interrupted aortic arch Type A, aortic stenosis, and coarctation of the aorta, for example) (Botto et al. 2007) receive more actionable recommendations, specifically for cardiac screening of first-degree relatives due to their high heritability, we hypothesize that parents of these infants will have higher empowerment and thus have higher subscale scores resulting in more positive Z-scores in their empowerment profiles. Similarly, we hypothesized that parents of infants with diagnostic rGS results would have higher empowerment and as a result have more positive scores in their empowerment profiles. This hypothesis was based on the idea that receiving a diagnostic result often comes with specific medical management recommendations. These may include investigating additional body systems through imaging, pursuing surgical interventions, starting medications, or initiating familial genetic testing. Additionally, knowing the underlying cause of their child’s heart defect (Musunuru et al. 2020), ) may provide parents with a greater sense of clarity and control, which could contribute to higher feelings of empowerment. Finally, we compared empowerment between parents of infants based on number of genetics appointments. We hypothesized that the more genetics encounters a parent had the more positive their empowerment will be due to the increased education and psychosocial support that is often provided at these encounters (Ison et al. 2019).

To our knowledge there have been no studies that assess empowerment of parents of infants with CHD following rGS. The aim of this study was to better understand the parental experience of having a critically ill child in the ICU through empowerment profiles, in addition to understanding the factors that influence empowerment in this parent population, all with the goal to better care for these families. We hypothesized that participants of infants with LVOTO CHDs, diagnostic rGS results, and/or more genetics visits will exemplify more positive Z-scores on corresponding empowerment profiles.

## Methods

This was a cross-sectional, quantitative study approved by the Indiana University (IU) School of Medicine Institutional Review Board (#22966).

### Participants and procedures

A list of eligible patients was derived from an internal clinical registry and the eligibility of each participant was verified by confirming at least one parent was English speaking and their child had completed rGS at less than 1 year of age between July 1, 2022 and May 31, 2024, via examination of the electronic medical record (EMR). All data was recorded in Research Electronic Data Capture (REDCap) (Harris et al. 2009, 2019). Additional information collected and/or confirmed from the EMR included name, date of birth, parent phone number, sex at birth, number of postnatal visits with a genetics provider, rGS results, mortality status of the infant, and level 3 Botto classification of the infant’s CHD (e.g., LVOTO, conotruncal, septal) (Botto et al. 2007).

**Fig. 1:**
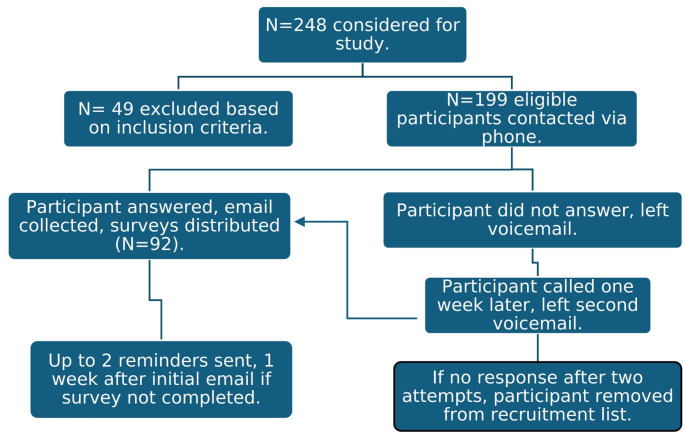
Participant recruitment

Due to institutional IRB requirements, eligible participants were first contacted by telephone to determine if they were interested in learning more about the study and willing to share their email address. Those who were able to be reached and agreed were sent an email with more information and a link to the survey. For those who could not be reached, a voicemail was left, and they were called again one week later if they did not respond to the initial voicemail. If after two attempts the participant could not be reached, they were removed from recruitment. Participants who were emailed a survey but did not complete it within 7 days were sent up to 2 automated REDCap email reminders, one week apart. This process, including number of participants ultimately sent a survey, is summarized in Fig. 1. All participants were initially contacted between July 9, 2024, and August 16, 2024. The study closed to participation on August 30, 2024, 2 weeks after the final reminder email was sent.

### Survey

The survey included questions to obtain demographic (level of education, age, gender, race, and ethnicity) and family-based (relationship of the parent/guardian to the child, primary medical coordinator status, the number of additional children under the care of the parent, and family history of CHD) information. The remaining questions consisted of the adapted GEmS (McConkie-Rosell et al. 2019). Nine questions (1, 7, 9, 11, 12, 14, 17, 18, 28) were altered slightly to reflect that rGS had already taken place, for example by changing words/phrases like “*will* you feel” to “*did* you feel.” This measure consists of four subscales: ‘Meaning of a Diagnosis’ (8 questions), ‘Emotional Management’ (8 questions), ‘Seeking Information and Support’ (7 questions), and ‘Implications and Planning’ (6 questions). The possible responses for each question were on a Likert scale of 1 = not important/well/certain/at all to 7 = very important/well/certain/much (see Appendix Fig. 1 for the survey codebook for additional details).

### Data analysis

The score for each subscale was obtained by summing the Likert values (1–7) for each participant’s response. A total empowerment score was then calculated by combining the scores within each subscale.

To standardize the sub-scales for data analysis and create the participant empowerment profiles, we converted the raw subscale scores of each participant into standardized Z-scores by subtracting the mean for each sub-scale and dividing by the standard deviation of the sub-scale. Each participant was coded as having a positive Z-Score or a negative Z-Score for each sub-scale to create an empowerment profile. This method was adapted from McConkie-Rosell et al., where the names for the empowerment profiles were also derived (i.e., Confident Realist, Resigned Acceptor) (McConkie-Rosell et al. 2022). Participant empowerment profiles were determined based on the pattern of positive or negative Z-Scores of each subscale (Table 1) in the following order: Meaning of a Diagnosis, Emotional Management, Seeking Information and Support, and Implications and Planning. Empowerment Profiles included ‘Disengaged or Overwhelmed’ (-, -, -, -), Resigned Acceptor (-, +, -, -), Confident Realist (+, +, +, -), and Engaged but Worried Planner (+, -, +, +), based on the analysis method of McConkie-Rosell et al. Additionally, we created empowerment profiles based on just the first two subscales (Meaning of a Diagnosis, Emotional Management) as they are more emotion-focused compared to the action scales (Seeking Information and Support, and Implications and Planning). Utilizing this method, which we call the Cheney method, we were able to categorize all participants into one of four categories based just on their emotion-focused subscales.

**Table 1:** Empowerment profile categorization based on gems subscale Z-scores

	Empowerment Profiles				
GEmS Subscales	Engaged but worried planner	Resigned Acceptor	Confident Realist	Disengaged or Overwhelmed	Uncategorized
McConkie Method					
**Meaning of a diagnosis**	+	-	+	-	Does not meet criteria for any of the four empowerment profiles
**Emotional Management**	-	+	+	-	
**Seeking Information and Support**	+	-	+	-	
**Implications and Planning**	+	-	-	-	
**Participant responses with empowerment profile***N* = *37(%)*	9(24)	6(16)	3(8)	3(8)	16(43)
Cheney Method					
**Meaning of a diagnosis**	+	-	+	-	N/A
**Emotional Management**	-	+	+	-	
**Seeking Information and Support**	+/-	+/-	+/-	+/-	
**Implications and Planning**	+/-	+/-	+/-	+/-	
**Participant responses with empowerment profile***N* = *37(%)*	15(40.5)	11(30)	6(16)	5(13.5)	0(0)

To further understand parental empowerment, we summarized parent empowerment profiles according to the following variables: LVOTO CHD versus non-LVOTO CHD, number of genetics visits, and rGS result type (diagnostic versus VUS or negative). To compare empowerment profiles of parents with infants who had a diagnostic rGS result with those that had a non-diagnostic result, negative and VUS results were both considered non-diagnostic. Lastly, we collapsed the number of postnatal genetics visits into three groups of approximately 30–40% each: those with 2 (*N* = 1) or 3 (*N* = 10) genetics visits (*N* = 11, 29.7%), those with 4 genetics visits (*N* = 14, 37.8%), and those with 5 (*N* = 7), 6 (*N* = 2), 7 (*N* = 2), or 11 (*N* = 1) visits (*N* = 12, 32.4%). No statistical analyses were performed due to small sample sizes in some comparison groups.

## Results

While it was the intent of this study to collect responses from multiple parents per infant, only two infants had completed surveys from more than one parent, therefore, only the first parent to respond was included in the analysis such that, while 39 responses were obtained 37 participant responses are analyzed. Although six participants did not complete much of the demographic portion of the survey, of the participants who did, 97% were female (*N* = 30), 90% self-identified as white (*N* = 33), and 65% reported having some post-secondary education (*N* = 20) as shown in Table 2.

**Table 2:** Demographics of parent participants

Participant/Parental Demographics	*N* (%)
Education*	
Higher education	20 (65)
High school education or less	11 (35)
Gender*	
Female	30 (97)
Male	1 (3)
**Race**	
Black or African American	2 (5)
White	33 (90)
Other/missing	2 (5)
Ethnicity*	
Hispanic/Latino	3 (10)
Non-Hispanic/Latino	23 (74)
Other ethnicity	5 (16)
Not pursued medical care due to finances*	
Yes	5 (16)
No	26 (84)
Primary Medical Coordinator*	
Myself	20 (65)
Someone else	11 (35)

*Six participants did not complete these questions, resulting in *N* = 31

Demographic and clinical information on the infants was collected from the EMR as shown in Table 3, in addition to self-reported information by participants (‘biological relative with a CHD’). The majority of participants had a child with a non-LVOTO CHD (*N* = 31, 84%), a negative result on rGS (*N* = 23, 62%) and did not have a biological relative with a CHD (*N* = 26, 84%). There was a roughly equal distribution in the count of the sex of the infant and the number of genetics visits between participants (Table 3).

**Table 3:** Demographics of infants with CHD

Infant Clinical Information	*N* (%)
**Sex**	
Female	18 (49)
Male	19 (51)
**Type of Heart Defect**	
LVOTO CHD	6 (16)
Non-LVOTO CHD	31 (84)
**Genetic Testing Result**	
Diagnostic result	9 (24)
Nondiagnostic result	28 (76)
Negative Result	23 (62)
VUS result	5 (14)
**Number of Genetics Visits**	
Three or more visits	11 (30)
Four visits	14 (38)
Five or more visits	12 (32)

CHD = congenital heart disease. LVOTO = left ventricular outflow tract obstruction

The GEmS subscale and total scores were calculated for each participant, and the summary statistics are reported in Table 4, specifically the range, mean, and standard deviation in addition to the minimum and maximum possible scores. This data was then utilized to calculate the Z-score for each participant for each subscale.

### GEmS empowerment raw scores

**Table 4:** Empowerment scores from the gems survey (*N* = 37)

Scale	Min-Max	Range	Mean	Std Dev
**Meaning of a Diagnosis**	8–56	21–54	43.2	8.1
**Emotional Management**	8–56	8–50	25.1	8.8
**Seeking Information and Support**	7–49	12–48	33.5	8.0
**Implications and Planning**	6–42	13–42	31.6	7.5
**Total GEmS**	29–203	82–163	133.5	19.5

gems = Genomic empowerment scale. Min-Max = minimum and maximum possible scores

**Fig. 2:**
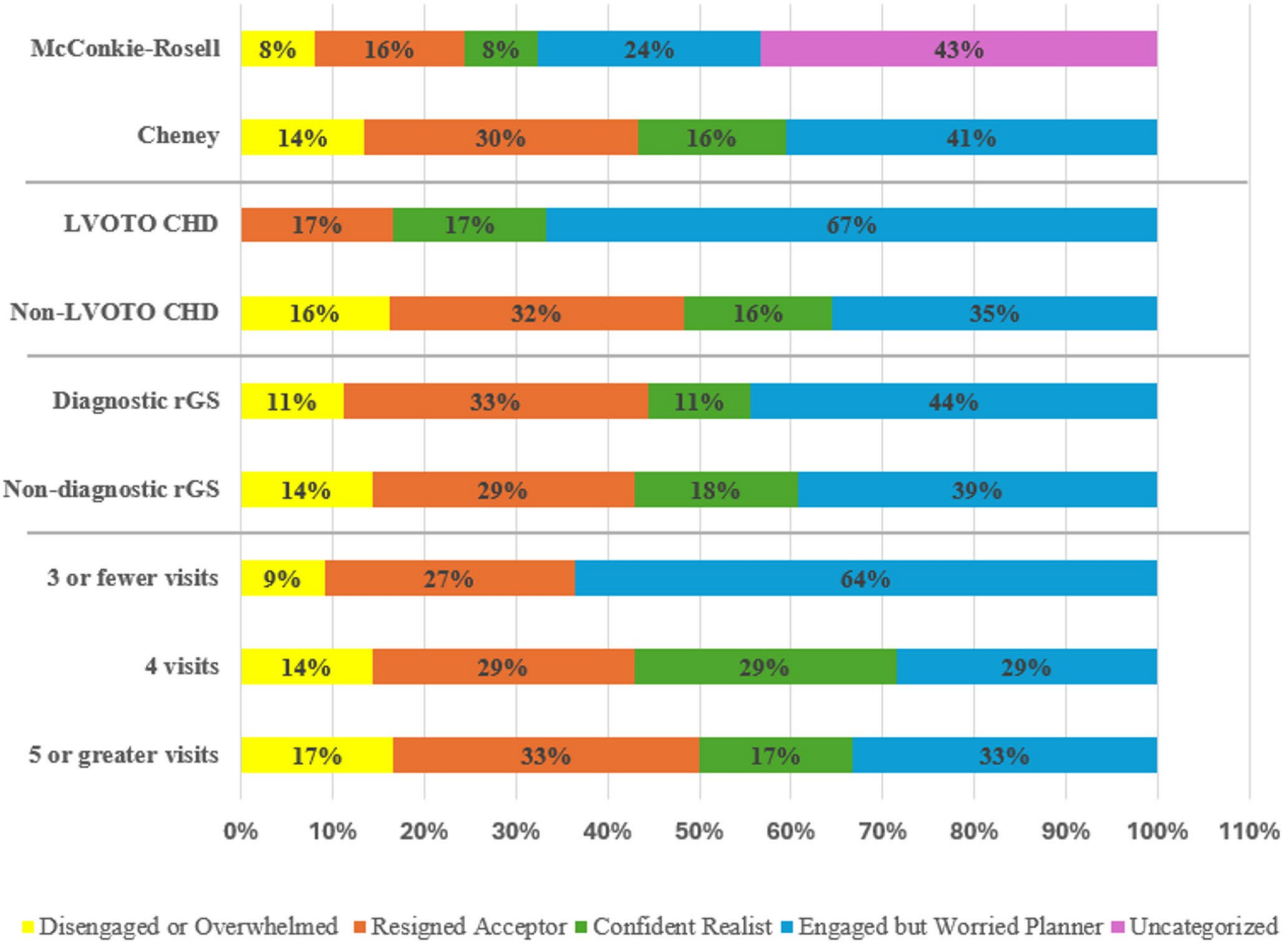
The percentage of each participant empowerment profile for each variable was calculated based on the number of participants in each variable group: McConkie-Rosell (*N* = 37), Cheney (*N* = 37), LVOTO CHD (*N* = 6), non-LVOTO CHD (*N* = 31), diagnostic rGS (*N* = 9), nondiagnostic rGS (*N* = 28), 3 or fewer visits (*N* = 11), 4 visits (*N* = 14), 5 or greater visits (*N* = 12). LVOTO = left ventricular outflow tract obstruction. rGS = rapid genome sequencing

Then, following the assignment of empowerment profiles using the McConkie-Rosell method of categorization, the distribution of profiles was such that the most common empowerment profile was the ‘Engaged but Worried Planner’ (*N* = 9, 24%), followed by the ‘Resigned Acceptor’ (*N* = 6, 16%) and three participants (8%) in each the ‘Confident Realist’ profile and ‘Disengaged or Overwhelmed’ profile. As nearly half of our respondents’ empowerment profiles did not fit the four empowerment profiles described in the McConkie-Rosell paper (*N* = 16, 43%) we applied the Cheney method, as described above, to enable categorization of all participants. These data are summarized in Table 1 and seen in Fig. 2.

### Participant empowerment profiles

The distribution of parental empowerment profiles categorized by the Cheney method differentiated by the variables of interest. Specifically, LVOTO CHD vs. non LVOTO CHD, diagnostic vs. nondiagnostic rGS result, and number of genetics visits is illustrated in Fig. 2.

## Discussion

To our knowledge, this is the first study to explore empowerment of parents whose infant with CHD had rGS in the neonatal/cardiovascular ICU. The results from this study demonstrate the diverse psychological status of parents in ICU settings through a lens of empowerment profiles. Our study found that nearly half of parents displayed the empowerment profile ‘Engaged but Worried Planner’, consistent with the most common empowerment profile among parents of children with undiagnosed disorders prior to genomic testing (McConkie-Rosell et al. 2022). As described by McConkie-Rosell et al., this profile type is consistent with parents in previous studies who have self-reported increased psychological distress and tend to neglect their own emotional well-being, while still expressing interest and engagement in their child’s medical care (Appendix, Table 1) (McConkie-Rosell et al. 2018, 2019, 2022). While inconsistent with our hypothesis, this empowerment profile aligns with previous literature that shows parents of infants with CHD often experience high emotional stress and ignore their own needs (Zhu et al. 2022). Additionally, it has been shown that attending to the needs of parents of children in the ICU can help to reduce anxiety in parents (Golfenshtein et al. 2019). These studies in combination with our results suggest that encouraging parents to prioritize taking care of themselves may be one avenue to help reduce the emotional burden of parents in this setting. Genetics providers should strive to categorize, by such methods as asking parents with whom they will have a long-term relationship to complete the GEmS survey or via collaboration with colleagues in social work and other areas who many already have discussed empowerment or similar topics with the parents, what type of profile the parent they are working with is in and increase psychosocial counselling to fill the gaps in areas of decreased empowerment for that profile type.

Interestingly, our results also aligned with the McConkie-Rosell study where the ‘Disengaged or Overwhelmed’ profile was the least common of the four profiles in their study (McConkie-Rosell et al. 2022). Parents with this profile scored below average in each subscale, highlighting that it is not common for parents to experience the feelings of disengagement from the diagnostic process or feelings of being overwhelmed (Table 1; Appendix, Table 1). This result is currently not well understood due to gaps in research, it is also possible that parents in this category are less likely to participate in studies and are not well represented in this work. It may be worthwhile to evaluate genetic testing/counselling pre- and post-test empowerment in a longitudinal study. However, genetics is only a small part of the medical experience of this population, and therefore it could be difficult to interpret the impact of genetic interaction in isolation from that of the entire care team.

In addition to the overall profile trends observed between our study and the McConkie-Rosell study, we explored if parents of infants with LVOTO CHDs displayed differences in empowerment profiles. We observed that parents of infants with LVOTO CHDs were nearly twice as likely to demonstrate the ‘Engaged but Worried Planner’ profile when compared to parents with infants with non-LVOTO CHDs; additionally, there were no participants with the ‘Disengaged or Overwhelmed’ profile in the LVOTO CHD cohort, whereas the non-LVOTO CHD cohort had a significant representation of this profile (*N* = 5, 16%). These trends in empowerment profiles may be explained by the increased management and screening recommendations made to infants with LVOTO CHDs based on the higher heritability of LVOTO CHDs (Shikany et al. 2019), possibly leading parents to feel increasingly engaged in the management and care of their infant.

We also explored an association between the category of rGS result (diagnostic versus nondiagnostic) and empowerment and found that there was little difference in empowerment profile distribution between groups. No matter the result type, most infants with critical CHD are offered surgical intervention and experience extended stays in ICU settings (Holst et al. 2017). Another study supports this notion, stating parents face emotional challenges in caring for their infant with critical CHD, often due to multiple surgeries, regardless of the result from genetic testing (Wei et al. 2015). Therefore, it is possible that result type does not lead to clear differences in empowerment profiles since most infants will experience similar courses of care, at least in the neonatal period, regardless of genetic etiology.

Finally, we sought out differences in empowerment profiles based on the number of genetics visits parents attended. Overall, there was a difference between three or less visits and four or more visits, however, the reason for this difference is unclear. There were more ‘Engaged but Worried Planners’ in the group with three or less visits, whereas participants with four or more visits displayed a more even distribution between the number of people in each empowerment profile (Fig. 2; Table 1 and Appendix, Table 1 for profile information). ‘Disengaged or Overwhelmed’ was also the least common profile between groups, consistent with the overall results from our study. Interestingly, no participants scored as ‘Confident Realists’ in the group with three or less visits. Given these results, the number of genetics visits has an unclear impact on empowerment and future research is warranted.

### Limitations and future directions

Due to institutional recruitment restrictions that limited access to the EMR to obtain contact information, specifically email addresses, the sample population for this study was especially limited as parents needed to be contacted via telephone and consent to the use of their email address. There may have been additional barriers to participation specific to genetics including that they have not followed up with genetics, or they didn’t remember receiving genetic testing/consultation well enough, or possibly not understanding the value of research participation. As a result, our parent population was small and rather homogeneous with enrichment of participants with higher levels of education. Homogeneity may also have directly influenced our data analysis as we calculated z-scores from the available data. Whatever the reason, future research should focus on reaching a larger and more diverse parent population to increase the generalizability of these results.

The McConkie-Rosell group designed their survey tool as a method of understanding the empowerment of parents prior to genetic testing. Given that our study administered the survey after the results of genetic testing were disclosed, our results should be interpreted from an exploratory perspective, as the survey tool was used outside of its intended purpose.

Healthcare empowerment is still in its infancy, therefore, there is still much work to be done in this area. Future research should focus on exploring the possible impact on empowerment in pre- and post-genetic testing cohorts in cardiovascular and other ICU settings for infants with CHD, as well as expanding analysis to larger hospital systems to increase understanding of empowerment in more diverse populations.

## Conclusion

As rGS continues to emerge as a first-tier diagnostic tool in infants with CHD, it is essential to also evaluate the psychosocial status of parents navigating this complex testing. Understanding the emotional, cognitive, and behavioral responses of parents following rGS can inform targeted interventions aimed at reducing distress and improving coping mechanisms. Furthermore, clarifying the role of genetics providers in this setting is crucial for optimizing support strategies and fostering parental empowerment. Increasing empowerment is particularly significant for parents of infants with CHD, as it may enhance aspects of their ability to comprehend their child’s diagnosis, engage in shared decision-making, and ability to advocate for appropriate medical care. By improving the care parents can give their infant, in addition to assisting in the coordination of their complex medical journey, healthcare teams can work toward improving the psychosocial well-being of parents and the overall clinical utility of rGS and genetics services in the ICU during the neonatal period.

## Electronic supplementary material

Below is the link to the electronic supplementary material.

Supplementary Material 1

Supplementary Material 2

## Data Availability

Data is available upon request from the authors.
