# Supplementary material for: Empowerment of parents of infants with congenital heart disease after rapid genome sequencing: April 11, 2025, revised June 20, 2025
Source: J Community Genet. 2025 Jul 14;16(5):619–27. doi: 10.1007/s12687-025-00813-3 (PMC12401776; doi:10.1007/s12687-025-00813-3)
Supplement: Supplementary file 1 — Supplementary Material 1 [file 12687_2025_813_MOESM1_ESM.pdf]

			1 Unverified<br>2 Complete
**Instrument: CHD Parent 1 Survey (chd_parent_1_survey)** Enabled as survey			
42	[consent]	Section Header: *Inclusion Criteria*<br>Do you agree to participate in this survey?	radio, Required<br>1 Yes<br>0 No<br>Stop actions on 0
43	[meaning_dx_label]	Section Header: *Survey Information Thank you for participating in this research study. We appreciate your time and perspective. Please read the information below and keep these things in mind while completing the survey. Please complete the survey in one sitting. In the questions below, when asked about your child's condition,please answer from the perspective regarding your child's specific heart defect.You will be asked to respond to questions related to your child's medical care, your family, and demographic information. This survey should take you approximately 15 minutes. In some of the questions below, genome sequencing is referenced. For clarity, you may have heard this term referred to as genetic testing, gene sequencing, and/or rapid genome sequencing during discussions with your medical providers. For the purpose of this study, all these terms should be interpreted with the same meaning.*<br>Meaning of a Diagnosis	descriptive
44	[meaning_of_dx_1]	1. Before you received the results from your child's genetic testing, how likely did you feel that the genetic sequencing would provide a diagnosis for your child?	radio, Required<br>1 1 not likely<br>2 2<br>3 3<br>4 4<br>5 5<br>6 6<br>7 7 very likely
45	[meaning_of_dx_2]	2. Do you feel that you can be an active partner with the medical genetics team in helping to find new information about your child?	radio, Required<br>1 1 not at all<br>2 2<br>3 3<br>4 4<br>5 5<br>6 6<br>7 7 very much
46	[meaning_of_dx_3]	3. How important do you feel a diagnosis is in making developmental therapy or educational	radio, Required<br>1 1 not important

		decisions for your child?	2 2<br>3 3<br>4 4<br>5 5<br>6 6<br>7 7 very important
47	[meaning_of_dx_4]	4. How important do you feel a diagnosis is in making medical decisions for your child?	radio, Required<br>1 1 not important<br>2 2<br>3 3<br>4 4<br>5 5<br>6 6<br>7 7 very important
48	[meaning_of_dx_5]	5. How important is it to you to have a diagnosis for your child's condition?	radio, Required<br>1 1 not important<br>2 2<br>3 3<br>4 4<br>5 5<br>6 6<br>7 7 very important
49	[meaning_of_dx_6]	6. How severe a problem do you view your child's condition?	radio, Required<br>1 1 not severe<br>2 2<br>3 3<br>4 4<br>5 5<br>6 6<br>7 7 very severe
50	[meaning_of_dx_7]	7. Do you feel that the information gained from the genetic sequencing has or will, in the future, improve your child's life?	radio, Required<br>1 1 not at all<br>2 2<br>3 3<br>4 4<br>5 5

			6 6<br>7 7 very much
51	[meaning_of_dx_expl_1]<br><br>Show the field ONLY if: [meaning_of_dx_7] = '1' or [meaning_of_dx_7] = '2' or [meaning_of_dx_7] = '3' or [meaning_of_dx_7] = '4' or [meaning_of_dx_7] = '5' or [meaning_of_dx_7] = '6'	If so, please list how?	notes
52	[meaning_of_dx_8]	8. How important is a genetic diagnosis for planning your child's future?	radio, Required<br>1 1 not important<br>2 2<br>3 3<br>4 4<br>5 5<br>6 6<br>7 7 very important
53	[emotional_label]	Emotional Management	descriptive
54	[emo_manage_1]	9. How do/did you think you would feel if the genetic sequencing did not provide a diagnosis for your child?	radio, Required<br>1 1 very negative<br>2 2<br>3 3<br>4 4<br>5 5<br>6 6<br>7 7 very positive
55	[emo_manage_2]	10. How emotionally difficult has the diagnostic process been so far?	radio, Required<br>1 1 not difficult<br>2 2<br>3 3<br>4 4<br>5 5<br>6 6<br>7 7 very difficult

56	[emo_manage_3]	11. Were you worried about what you might learn about your child's diagnosis from the genetic sequencing?	radio, Required<br>1	1 not worried<br>2	2<br>3	3<br>4	4<br>5	5<br>6	6<br>7	7 very worried
57	[emo_manage_expl_1]<br>Show the field ONLY if:<br>[emo_manage_3] = '1' or [emo_manage_3] = '2' or [emo_manage_3] = '3' or [emo_manage_3] = '4' or [emo_manage_3] = '6'	If so, what makes you worry? Please describe.	notes
58	[emo_manage_4]	12. Were you worried about new information you might learn from the genetic sequencing that is unrelated to your child's condition (reason the genetic sequencing is being done)?	radio, Required<br>1	1 not worried<br>2	2<br>3	3<br>4	4<br>5	5<br>6	6<br>7	7 very worried
59	[emo_manage_5]	13. How emotionally difficult would it be to handle uncertainty related to a diagnosis?	radio, Required<br>1	1 not difficult<br>2	2<br>3	3<br>4	4<br>5	5<br>6	6<br>7	7 very difficult
60	[emo_manage_6]	14. How confident did you feel that you will be able to emotionally manage whatever is learned from the genetic sequencing?	radio, Required<br>1	1 not confident<br>2	2<br>3	3<br>4	4

			5 5<br>6 6<br>7 7 very confident
61	[emo_manage_7]	15. How emotionally difficult would it be to handle a genetic diagnosis for which little information is known?	radio, Required<br>1 1 not difficult<br>2 2<br>3 3<br>4 4<br>5 5<br>6 6<br>7 7 very difficult
62	[emo_manage_8]	16. How physically difficult (e.g. clinic appointments, tests, hospitalizations etc.) has the diagnostic process been for your child?	radio, Required<br>1 1 not difficult<br>2 2<br>3 3<br>4 4<br>5 5<br>6 6<br>7 7 very difficult
63	[seek_info_support_label]	Seeking Information and Support	descriptive
64	[seek_info_support_1]	17. How confident are you that, if needed, you would be able to find assistance for your child to help with a new medical or developmental concern identified by the genetic sequencing?	radio, Required<br>1 1 not confident<br>2 2<br>3 3<br>4 4<br>5 5<br>6 6<br>7 7 very confident
65	[seek_info_support_2]	18. How likely do you feel it is that you would be able to find other families whose children have the same condition as your child?	radio, Required<br>1 1 not likely<br>2 2<br>3 3<br>4 4<br>5 5<br>6 6

			7 | 7 very likely
66	[seek_info_support_3]	19. Do you feel that you can learn helpful information from other families whose children have the same condition as your own child?	radio, Required<br>1 | 1 not at all<br>2 | 2<br>3 | 3<br>4 | 4<br>5 | 5<br>6 | 6<br>7 | 7 very much
67	[seek_info_support_4]	20. How important is it to you to find other families whose children have the same condition as your child?	radio, Required<br>1 | 1 not important<br>2 | 2<br>3 | 3<br>4 | 4<br>5 | 5<br>6 | 6<br>7 | 7 very important
68	[seek_info_support_5]	21. Are you confident in your ability to use the information from the genetic sequencing to help your child?	radio, Required<br>1 | 1 not confident<br>2 | 2<br>3 | 3<br>4 | 4<br>5 | 5<br>6 | 6<br>7 | 7 very confident
69	[seek_info_support_6]	22. Do you feel that you can use your child's genetic sequencing result to find new information on your own?	radio, Required<br>1 | 1 not at all<br>2 | 2<br>3 | 3<br>4 | 4<br>5 | 5<br>6 | 6<br>7 | 7 very much
70	[seek_info_support_7]	23. How well do you feel that you can understand the different types of genetic results that come from genetic sequencing?	radio, Required<br>1 | 1 not well<br>2 | 2

			3 3<br>4 4<br>5 5<br>6 6<br>7 7 very well
71	[implication_planning_label]	Implications and Planning	descriptive
72	[imp_plan_1]	24. How important is it for you to have a diagnosis for making decisions about having children?	radio, Required<br>1 1 not important<br>2 2<br>3 3<br>4 4<br>5 5<br>6 6<br>7 7 very important
73	[imp_plan_2]	25. How important is it for your family members who may be making decisions about having children to have a diagnosis for your child?	radio, Required<br>1 1 not important<br>2 2<br>3 3<br>4 4<br>5 5<br>6 6<br>7 7 very important
74	[imp_plan_3]	26. How important do you feel a diagnosis is for other families who are making decisions about having children?	radio, Required<br>1 1 not important<br>2 2<br>3 3<br>4 4<br>5 5<br>6 6<br>7 7 very important
75	[imp_plan_4]	27. How responsive has your child's local primary medical provider been to your concerns about your child's disorder?	radio, Required<br>1 1 not responsive<br>2 2<br>3 3<br>4 4

			5 5<br>6 6<br>7 7 very responsive
76	[imp_plan_5]	28. How much has your child's local primary medical provider partnered with you to make a plan for your child's care once you have a genetic sequencing result?	radio, Required<br>1 1 not at all<br>2 2<br>3 3<br>4 4<br>5 5<br>6 6<br>7 7 very much
77	[imp_plan_6]	29. How certain do you feel that you know what the "next steps" are for the management of your child's condition?	radio, Required<br>1 1 not certain<br>2 2<br>3 3<br>4 4<br>5 5<br>6 6<br>7 7 very certain
78	[primary_med_coordinator]	Section Header: *Additional Information About Your Family and the Care of Your Child*<br>Who is the primary medical coordinator in your family regarding your child's heart defect and medical management?	radio<br>1 Myself<br>2 Other Parent<br>3 Shared with Other Parent<br>4 Other
79	[med_coordinator_other]<br>Show the field ONLY if: [primary_med_coordinator] = '4'	Primary Medical Coordinator: 'Other'<br>*please specify 'other' medical care coordinator*	text
80	[realtionship_to_child]	What is your relationship to your child?	radio<br>1 Biological Parent<br>2 Adoptive Parent (or similar)<br>3 Foster Parent (or similar)<br>4 Other
81	[relationship_other]	Relationship to child: 'Other'	text

	Show the field ONLY if: [realtionship_to_child] = '4'		
82	[number_of_kids]	How many kids (18 years old or younger) are in your household? Please include the child for which you answered regarding this survey.<br>*number only*	text (number, Min: 1, Max: 20)
83	[relative_chd]	Does your child have another biological/blood relative with a heart defect?	radio<br>1 Yes<br>0 No
84	[relative_chd_type]<br>Show the field ONLY if: [relative_chd] = '1'	If so, please enter the type of heart defect of your relative here:	text
85	[rec_med_care]	Have you ever not pursued recommended medical care due to financial reasons?	radio<br>1 Yes<br>0 No
86	[education_level]	Section Header: *DemographicsPlease answer the following questions regarding yourself and NOT your child.*<br>What is your highest level of education?	radio<br>1 Partial High School or Less<br>2 High School Graduate<br>3 Some College<br>4 College Graduate<br>5 Graduate Degree
87	[parental_age]	How old are you?<br>*number only*	text (number, Min: 0, Max: 100)
88	[parental_gender]	What is your gender?	radio<br>1 Male<br>2 Female<br>3 Non-binary<br>4 Transgender<br>5 Other
89	[parent_gender_other]<br>Show the field ONLY if: [parental_gender] = '5'	Parental Gender: 'Other'	text (alpha_only)
90	[race]	Race (select all that apply)	checkbox<br>0 race___0 White

			1 race___1 Native American Indian or Alaska native<br>2 race___2 Asian<br>3 race___3 Native Hawaiian or other Pacific Islander<br>4 race___4 Black or African American<br>5 race___5 Prefer not to say<br>99 race___99 Other
91	[race_other]<br>Show the field ONLY if:<br>[race(99)] = "1"	Race: 'other'	text (alpha_only)
92	[ethnicity]	Ethnicity	radio<br>0 Non-hispanic/Latino<br>1 Hispanic/Latino<br>2 Ashkenazi Jewish<br>3 Other<br>99 Not reported/Unknown
93	[other_ethnicity]<br>Show the field ONLY if:<br>[ethnicity] = 'other'	Ethnicity: 'other'	text (alpha_only)
94	[secondary_email]	Section Header: *Additional Parent/Caregiver*<br>As we are trying to obtain as many parental perspectives as possible, we are wondering if your child has another parent/caregiver who would be willing to get an email about this study. If so, please provider their email address here.	text (email)
95	[gift_card_entry]	Section Header: *Prize Drawing*<br>Would you like to enter a free prize drawing for a $50.00 VISA gift card?	radio<br>1 Yes<br>0 No
96	[chd_parent_1_survey_complete]	Section Header: *Form Status*<br>Complete?	dropdown<br>0 Incomplete<br>1 Unverified<br>2 Complete
**Instrument: CHD Parent 2 Survey (chd_parent_2_survey)** **Enabled as survey**			
97	[consent_p2]	Section Header: *Inclusion Criteria*	radio, Required
