# Supplementary material for: Empowerment of parents of infants with congenital heart disease after rapid genome sequencing: April 11, 2025, revised June 20, 2025
Source: J Community Genet. 2025 Jul 14;16(5):619–27. doi: 10.1007/s12687-025-00813-3 (PMC12401776; doi:10.1007/s12687-025-00813-3)
Supplement: Supplementary file 2 — Supplementary Material 2 [file 12687_2025_813_MOESM2_ESM.docx]

**Appendix**

| Empowerment Profile | This Study | McConkie-Rosell, 2022 | High Stress | Engaged in Own Care | Engaged in Child’s Care |
| --- | --- | --- | --- | --- | --- |
| Engaged/Worried | Most Common | Most Common | Yes | No | Yes |
| Disengaged/Overwhelmed | Least Common* | Least Common* | Yes | No | No |

Table 1. Characteristics of the most and least common empowerment profiles. * Parents in this category may be less likely to participate in research studies.
